# Two genomes, one cell: Mitochondrial-nuclear coordination via epigenetic pathways

**DOI:** 10.1016/j.molmet.2020.01.006

**Published:** 2020-02-15

**Authors:** Meike Wiese, Andrew J. Bannister

**Affiliations:** 1 Max-Planck-Institute for Immunobiology und Epigenetics, Department of Chromatin Regulation, Stübeweg 51, 79108, Freiburg im Breisgau, Germany; 2 Gurdon Institute and Department of Pathology, University of Cambridge, Tennis Court Road, Cambridge, CB2 1QN, UK

**Keywords:** Epigenetics, Metabolites, Enzymes, RNA modification, Chromatin, Histones, Mitochondria

## Abstract

**Background:**

Virtually all eukaryotic cells contain spatially distinct genomes, a single nuclear genome that harbours the vast majority of genes and much smaller genomes found in mitochondria present at thousands of copies per cell. To generate a coordinated gene response to various environmental cues, the genomes must communicate with each another. Much of this bi-directional crosstalk relies on epigenetic processes, including DNA, RNA, and histone modification pathways. Crucially, these pathways, in turn depend on many metabolites generated in specific pools throughout the cell, including the mitochondria. They also involve the transport of metabolites as well as the enzymes that catalyse these modifications between nuclear and mitochondrial genomes.

**Scope of review:**

This study examines some of the molecular mechanisms by which metabolites influence the activity of epigenetic enzymes, ultimately affecting gene regulation in response to metabolic cues. We particularly focus on the subcellular localisation of metabolite pools and the crosstalk between mitochondrial and nuclear proteins and RNAs. We consider aspects of mitochondrial-nuclear communication involving histone proteins, and potentially their epigenetic marks, and discuss how nuclear-encoded enzymes regulate mitochondrial function through epitranscriptomic pathways involving various classes of RNA molecules within mitochondria.

**Major conclusions:**

Epigenetic communication between nuclear and mitochondrial genomes occurs at multiple levels, ultimately ensuring a coordinated gene expression response between different genetic environments. Metabolic changes stimulated, for example, by environmental factors, such as diet or physical activity, alter the relative abundances of various metabolites, thereby directly affecting the epigenetic machinery. These pathways, coupled to regulated protein and RNA transport mechanisms, underpin the coordinated gene expression response. Their overall importance to the fitness of a cell is highlighted by the identification of many mutations in the pathways we discuss that have been linked to human disease including cancer.

## Highlights

• Communication between mitochondria and nuclei coordinates epigenetic processes regulating a cell's response to external cues
• Mitochondrial metabolism mediates the nuclear epigenome through trafficking of metabolic enzymes and metabolites to the nucleus
• Subcellular localisation and abundance of metabolites tightly control activity of epigenetic and epitranscriptomic enzymes
• Nuclear epigenetic enzymes and RNAs translocate to the mitochondria and regulate mitochondrial metabolism
• Modifications of mitochondrial RNAs mediated by nuclear encoded epitranscriptomic enzymes regulate mitochondrial function

## 1 Introduction

To fit within the confined space of a eukaryotic nucleus, a cell's genome must be efficiently compacted, but in a highly ordered manner that maintains accessibility to the genetic information. This is achieved by DNA complexing with histone proteins to form nucleosome structures, which in turn further compact to form chromatin that ultimately packages entire chromosomes. Importantly, chromatin is not an inert packaging structure but rather an instructive scaffold capable of responding to various cues to regulate access of the DNA to different cellular machineries. This accessibility is fundamentally regulated by ATP-dependent chromatin remodelling activities and by covalent modification of both DNA and histone proteins. These modifications are commonly referred to as “epigenetic” modifications. Furthermore, in addition to epigenetics, the emerging field of epitranscriptomics is now adding numerous modifications of RNA molecules to the “modification repertoire”, thereby expanding our current view of epigenetics and how it regulates all DNA processes, in particular gene expression [1].

DNA and histone modifications regulate gene expression by modulating chromatin accessibility and/or providing binding sites for regulatory proteins. To date, more than 100 post-translational modifications (PTMs) have been detected on histones, including acetylation, methylation, ubiquitylation, phosphorylation, citrullination, and O-GlcNAcylation. Of these, acetylation and methylation are by far the best-studied and most characterised (for review, see [2,3]). The establishment of epigenetic signatures is accomplished by enzymes that write or erase different types of PTMs on specific histone amino acids [2,3] or modify DNA by introducing or removing a methyl group at the 5’ position of cytosines [4]. The same types of enzymes also dynamically modify RNA molecules at specific internal nucleotides, with methylation again being by far the most prevalent modification (for review, see [5]).

**Figure 1:**
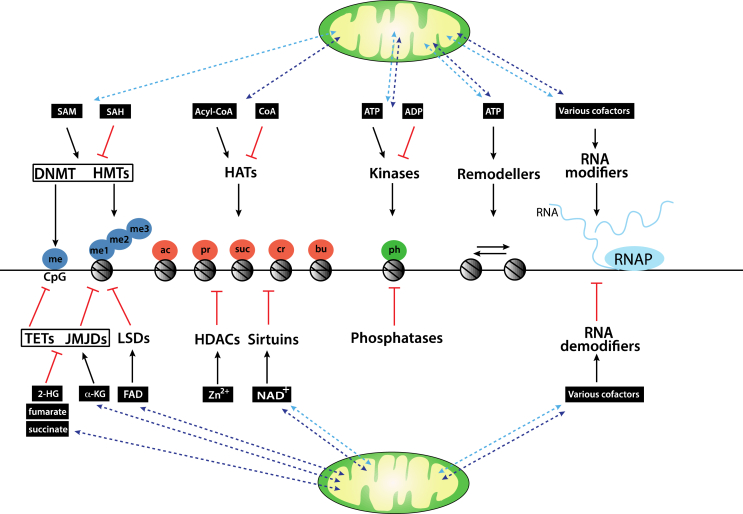
*Metabolic intermediates impact chromatin modifications*. Epigenetic modifiers use metabolites as cofactors that are either directly produced within mitochondria (dark blue dotted arrows) or their production is indirectly affected by metabolic pathways within mitochondria (light blue dotted arrows). HMTs and DNMTs use SAM as a methyl donor to methylate (me) histones at lysine or arginine residues, or DNA, respectively. Both enzyme families are inhibited by the by-product of the methylation reaction, SAH. TET DNA dioxygenases and HDMs of the JMJD family use the tricarboxylic acid cycle intermediate α-KG as a cofactor and are inhibited by fumarate, succinate, and 2-HG. HDMs of the lysine-specific histone demethylase (LSDs) family use the cofactor FAD. HATs can utilise acyl-CoA derivatives acetyl (ac)-CoA, propionyl (pr)-CoA, succinyl (suc)-CoA, crotonyl (cr)-CoA, or butyryl (bu)-CoA as cofactors. Some HATs are inhibited by the acetylation by-product CoA. Histone acetylations are removed by HDACs. The sirtuin family of HDACs uses NAD^+^ as a cofactor, which is generated in the mitochondria, cytoplasm, and nucleus. Other HDACs depend on Zn^2+^, which is generated ubiquitously. Histones can be phosphorylated (ph) at serine, threonine, or tyrosine residues by ATP-dependent kinases that are inhibited by the by-product ADP. Dephosphorylation is mediated by phosphatases. Mitochondrially generated ATP is an essential cofactor for ATP-dependent remodelling activities that use the energy derived from ATP hydrolysis to move/slide nucleosomes. There are many families of RNA-modifying (and de-modifying) enzymes that require a wide variety of cofactors for their catalytic activity (see the text for details). *Abbreviations*: 2-HG: 2-hydroxyglutarate, α-KG: α-ketoglutarate, Acyl-CoA: acyl-coenzyme A, ADP: adenosine diphosphate, ATP: adenosine triphosphate, DNMT: DNA methyltransferase, FAD: flavin adenine dinucleotide, HAT: histone acetyltransferase, HDAC: histone lysine deacetylase, HDM: histone demethylase, HMT: histone methyltransferase, JMJD: Jumonji C domain-containing protein, LSD: lysine-specific histone demethylase, NAD^+^: nicotinamide adenine dinucleotide, SAH: S-adenosyl-homocysteine, SAM: S-adenosyl methionine, TET: ten-eleven translocation. (For interpretation of the references to colour in this figure legend, the reader is referred to the Web version of this article).

Regulating cell metabolism to support all instrumental cellular activities is essential for the maintenance of cell homeostasis, growth, proliferation, migration, differentiation, and apoptosis. The regulation of cell metabolism involves coordinated changes in gene expression in response to extracellular and intracellular signals to manage the needs of cells and adapt to environmental flux. It is now apparent that the abundance of certain metabolites can be drastically affected by alterations in diet, as well as more general environmental perturbations. In turn, these changes can have profound consequences on mitochondrial and nuclear functions. Metabolic intermediates can, directly or indirectly, impact chromatin modifications and thereby influence chromatin functions. Histone, DNA, RNA modifications, and many chromatin remodelling activities strictly depend on the availability of metabolites, which are essential cofactors for chromatin and RNA-modifying enzymes (Figure 1). Thus, metabolism is closely integrated with epigenetic and epitranscriptomic processes to regulate physiological responses to extracellular stimuli, and deregulation of these interconnected pathways can have profound consequences, leading to the development of specific diseases, including cancer.

Mitochondrial activity underpins all metabolic processes. Organelles generate sufficient adenosine triphosphate (ATP) for most cellular functions, including chromatin remodelling activities, via oxidative phosphorylation (OXPHOS), a multistep process that oxidises hydrogens to generate water and release energy [6], which accounts for approximately 90% of the body's total energy production. The energy, released as electrons, shuttles through the electron-transport chain (complexes I, III, and IV) and is finally used to pump protons across the mitochondrial inner membrane. In this way, an electrochemical gradient is generated that subsequently drives ATP synthase (complex V) to create ATP from ADP and phosphate [6]. Mitochondria also house the fatty acid (FA) oxidation machinery, which is the main source of acetyl-coenzyme A (acetyl-CoA), an important metabolite for epigenetic modifications, at least in mammals. Thus, mitochondrial activity is intricately coupled to epigenetic and epitranscriptomic processes.

**Figure 2:**
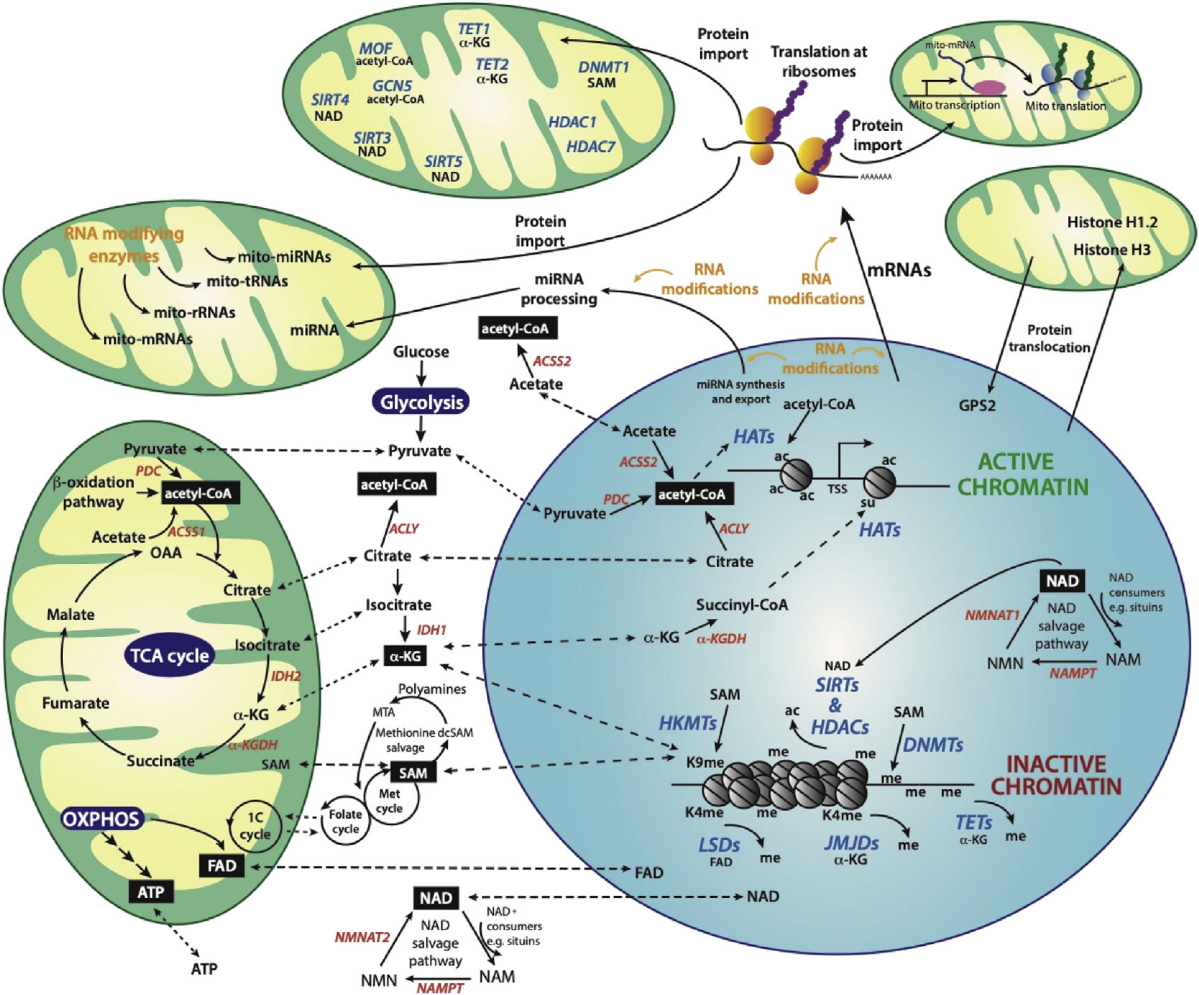
*Epigenetic and epitranscriptomic pathways depend on metabolism*. Crosstalk between the mitochondria and nucleus is regulated by the translocation of metabolic enzymes, metabolites, RNAs, and epigenetic regulators. Epigenetic gene regulation is established by enzymes that write, read, or erase post-translational modifications on histones, in particular acetylation (ac) and methylation (me), and regulate the methylation of DNA. In a similar fashion, modifications of RNA molecules have the potential to regulate all RNA-dependent functions and activities. Acetyl-CoA, the donor of acetyl groups used by HATs, is generated in distinct pools within the cell. Three main pathways in the mitochondria generate acetyl-CoA: (i) fatty acid breakdown via the β-oxidation pathway, (ii) PDC acting on pyruvate derived from glucose through glycolysis, and (iii) ACSS1 acting on acetate. Mitochondrial acetyl-CoA is subsequently used to acetylate mitochondrial proteins or channelled into the TCA cycle to generate energy. In the cytoplasm, two main pathways generate acetyl-CoA: (i) by ACLY acting on citrate, which in turn can be generated from the TCA cycle and shuttled between the mitochondria and cytoplasm and (ii) by ACSS2 on acetate. The three main precursors of acetyl-CoA (pyruvate, acetate, and citrate) can also diffuse into the nucleus, where they are processed into acetyl-CoA by nuclear pools of PDC, ACSS2, and ACLY, respectively. Distinct pools of acetyl-CoA regulate acetylation of mitochondrial, cytoplasmic, and nuclear proteins, including histones. HAT-mediated histone acetylation results in chromatin de-condensation and active transcription (active chromatin). Nuclear HATs MOF and GCN5 also localise to mitochondria. For more information on nuclear epigenetic factors localising to mitochondria (blue), see the main text and Table 1. α-KG is generated in the TCA cycle in mitochondria from isocitrate by IDH2. Isocitrate can diffuse into the cytosol where it is converted by IDH1 to α-KG. α-KG diffuses into the nucleus, where it is used as a cofactor by JMJD histone demethylases, TET DNA demethylases, or is converted by α-KGDH into succinyl-CoA, a cofactor for HATs. Mitochondria regulate the redox levels of FAD, a cofactor for LSDs. During β-oxidation and the TCA cycle, FAD is reduced to FADH_2_ and returned to its oxidised form during OXPHOS. FAD can then diffuse into the cytosol and nucleus. SAM is the donor of methyl groups utilised by HMTs and DNMTs in the nucleus. SAM is generated through the coupling of the folate and methionine cycles in the cytosol, which in turn is sustained by 1-C metabolism in mitochondria or is generated via the methionine salvage pathway. SAM can also enter the mitochondria. Methylation of DNA and lysine (K) 9 of histone H3 (K9me) together with histone deacetylation are associated with chromatin compaction and gene repression (inactive chromatin). Sirtuin histone deacetylates use NAD^+^ as a cofactor, which is generated *de novo* from the amino acid tryptophan or via the NAD^+^ salvage pathway, which uses NAD^+^ precursors such as NAM directly from the diet or recycled from intracellular reactions. A nuclear pool of NAD^+^ is either generated via a nuclear NAD^+^ salvage pathway or through passive diffusion of cytoplasmic NAD^+^ through nuclear pores. All classes of nuclear RNAs (mRNAs, miRNAs, lncRNAs, rRNAs, and tRNAs) are transcribed from active chromatin and actively transported into the cytoplasm. miRNAs are processed in the cytoplasm and can be transported into the mitochondria, while mRNAs are translated at cytoplasmic ribosomes. Transcription in the mitochondria of RNAs (mRNAs, rRNAs, tRNAs, and perhaps miRNAs) also occurs with translation of the mRNAs at mito-ribosomes. Throughout the cell, all classes of RNAs are targeted by specific RNA-modifying enzymes (orange). Proteins synthesised by cytoplasmic ribosomes either reside in the cytoplasm, are imported into mitochondria and/or into the nucleus. During mitochondrial stress, mitochondrial GPS2 rapidly translocates to the nucleus where it collaborates with H3K9 demethylases to activate gene expression facilitating mitochondrial stress response. Also, certain nuclear proteins translocate to the mitochondria only during cellular stress. For example, both histone H3 and H1.2 are released from chromatin and translocate to the mitochondria during apoptosis. This figure was adapted from [145]. *Abbreviations*: 1-C: one-carbon, Acetyl-CoA: acetyl-coenzyme A, ACLY: ATP-citrate lyase, ACSS1: acyl-CoA synthetase short-chain family member 1, α-KG: α-ketoglutarate, α-KGDH: α-ketoglutarate dehydrogenase, ATP: adenosine triphosphate, dcSAM: decarboxylated SAM, DNMT: DNA methyltransferase, FAD: flavin adenine dinucleotide, GCN5: lysine acetyltransferase 2A, GPS2: G-protein pathway suppressor 2, HAT: histone acetyltransferase, HDAC: histone lysine deacetylase, HMT: histone methyltransferase, IDH2/3: isocitrate dehydrogenase 2/3, JMJD: Jumonji C domain-containing histone demethylase, K9: lysine 9, LSDs: lysine-specific histone demethylases, MOF: lysine acetyltransferase 8, MTA: 5′-methylthioadenosine, NAD^+^: nicotinamide adenine dinucleotide, NAM: nicotinamide, NAMPT: nicotinamide phosphoribosyl transferase, NMN: nicotinamide mononucleotide, NMNAT1/2: nicotinamide mononucleotide adenylyltransferase 1/2/3, OAA: oxaloacetate, OXPHOS: oxidative phosphorylation, PDC: pyruvate dehydrogenase complex, SAM: *S*-adenosyl methionine, SIRT: sirtuin deacetylase, TCA: tricarboxylic acid, TET: ten-eleven translocation DNA demethylase. (For interpretation of the references to colour in this figure legend, the reader is referred to the Web version of this article).

The mitochondrial genome is maternally inherited and encodes only 13 polypeptide genes, whereas the nuclear DNA (nDNA) harbours thousands of genes, including those encoding structural mitochondrial proteins and enzymes that maintain mitochondrial DNA (mtDNA). This situation underpins the concept of two genomes, one cell, which holds that eukaryotic cells contain genetic material in two distinct compartments, the nucleus and mitochondria. It then follows that these two genetic compartments must be co-ordinately controlled to regulate both nuclear and mitochondrial functions. For instance, most polypeptides required for mitochondrial OXPHOS are encoded within the nuclear genome, strongly implying that coordinated expression of mitochondrial and nuclear genes is indispensable. Such coordination is achieved through bidirectional exchange of polypeptides, enzymes, and metabolites between the two compartments (Figure 2).

However, the situation is more complex than implied by the simple two genomes, one cell concept. This is because mitochondria are present in thousands of copies per cell and each mitochondrion on has autonomous multi-copy, double-stranded circular DNA genomes. Furthermore, mtDNA has a much higher mutation rate than nDNA, meaning that a single cell usually contains many mitochondrial genomes that differ to some degree at the nucleotide sequence level (heteroplasmy). Heteroplasmy and the interplay between the nuclear and mitochondrial genomes might explain the transmission of certain complex diseases, such as metabolic syndromes, neurodegenerative diseases, or cancer, that cannot be explained by Mendelian laws of inheritance and that are strongly influenced by environmental factors [6]. For example, the highly pathogenic tRNA^Leu(UUR)^ m.3243 A>G point mutation is one of many disease-associated mtDNA mutations [6] that cause different clinical phenotypes depending on the level of mutated mtDNA [7]. While patients with a relatively low level of heteroplasmy (20–30% of m.3243G mutant mitochondria) develop diabetes, those with higher heteroplasmy show increasingly more severe phenotypes, such as cardiomyopathy and stroke-like symptoms, and ultimately death (90–100% heteroplasmy).

The gradual change in phenotype severity due to increasing m.3243G mutant mitochondria heteroplasmy was recently linked to distinct alterations in mitochondrial metabolic pathways, which in turn were associated with remodelling of the nuclear epigenome [7]. More specifically, changes in mtDNA heteroplasmy induce changes in mitochondrial metabolites and redox state, which results in specific alterations of histone modifications and transcriptional changes in both nDNA and mtDNA [7]. These findings highlight the importance of mitochondrial and nuclear crosstalk in cellular homeostasis and emphasis how small changes in the mitochondrial genome can completely rewire the metabolic status of a cell through epigenetic processes [7].

Many mitochondria-encoded RNAs are also modified in the mitochondria by nuclear-encoded enzymes, which also function in the nucleus. Many other epigenetic enzymes that have well-characterised nuclear chromatin functions are also found in the mitochondria. A schematic overview of the principles of communication between nuclei and mitochondria discussed in this review are illustrated in Figure 2. There is emerging evidence that various nuclear-encoded metabolic enzymes, which typically localise to the cytoplasm or mitochondria, are also present in the nucleus (Table 1). This dynamic localisation of metabolic enzymes regulates metabolite levels in a spatiotemporal manner with direct consequences on the epigenetic regulation of gene expression.

**Table 1:** Crosstalk between mitochondrial and nuclear enzymes affecting epigenetic pathways. (A) Metabolic enzymes predominantly generating metabolic intermediates in the mitochondria (mt) or cytoplasm (cyt) regulate epigenetic processes in the nucleus (nuc). **(B)** Epigenetic enzymes utilising metabolic intermediates have well-defined nuclear functions, translocate to mitochondria, and affect mitochondrial function.

A				
Metabolic enzyme	Metabolic produced	Intracellular localisation	Epigenetic effect	Reference
PDC	acetyl-CoA	mt, nuc	production of acetyl-CoA for histone acetylation (nuc)	[29,30]
Citrate synthetase	citrate	mt, nuc	production of citrate as a precursor for acetyl-CoA used for histone acetylation (nuc)	[30]
AC02	isocitrate	mt, nuc	production of isocitrate as a precursor for acetyl-CoA used for histone acetylation (nuc)	[30]
IDH3	α-KG	mt, nuc	activation of α-KG-utilising DNA and histone demethylases (mt, nuc)	[30]
α-KGDH	succinyl-CoA	mt, nuc	production of succinyl-CoA for KAT2A mediated histone succinylation (nuc)	[34]
Fumarase	malate	mt, nuc	inhibition of α-KG-utilising DNA and histone demethylases (mt, nuc)	[89]
GDH1	α-KG	mt, nuc	activation of α-KG-utilising DNA and histone demethylases (mt, nuc), H3 tail clipping (nuc)	[67,68]
ACSS2	acetyl-CoA	cyt, nuc	production of acetyl-CoA for histone acetylation (nuc)	[27,28]
ACLY	acetyl-CoA	cyt, nuc	production of acetyl-CoA for histone acetylation (nuc)	[[20], [21], [22], [23]]

B				
Epigenetic enzyme	Metabolic utilised	Intracellular localisation	Epigenetic effect	Reference

MOF	acetyl-CoA	mt, nuc	histone acetylation (nuc), regulation of mtDNA transcription (mt), OXPHOS regulation (mt)	[36]
GCN5	acetyl-CoA	mt, nuc	histone acetylation (nuc), mt function unknown	[40]
SIRT3	NAD^+^	mt, nuc	deacetylation of metabolic enzymes (mt), histone and TF deacetylation (nuc)	[[54], [55], [56], [57], [58], [59]]
HDAC1	Zn^2+^	mt, nuc	histone deacetylation (nuc), mt function unknown	[41]
HDAC7	Zn^2+^	mt, nuc, cyt	histone deacetylation (nuc), mt function unknown	[42]
DNMT1	SAM	mt, nuc	maintenance DNA cytosine methylation (nuc), mt function unknown	[82]
TET1/2	α-KG	mt, nuc	Methylcytosine dioxygenation of DNA (nuc), mt function unknown	[96]

*Abbreviations*: α-KGDH: α-ketoglutarate dehydrogenase, ACLY: ATP-citrate lyase, ACO2: aconitase 2, ACSS2: acyl-CoA synthetase short-chain family member 2, DNMT1: DNA methyltransferase 1, IDH3: isocitrate dehydrogenase 3, GCN5: lysine acetyltransferase 2A, GDH1: glutamate dehydrogenase, HDAC1 and 7: histone lysine deacetylase 1 and 7, MOF: lysine acetyltransferase 8, PDC: pyruvate dehydrogenase complex, SIRT3: sirtuin deacetylase 3, TET1/2: ten-eleven translocation DNA demethylase 1/2.

This review describes some of the molecular mechanisms by which metabolites influence the activity of epigenetic enzymes, ultimately affecting gene regulation in response to metabolic cues (Sections 2, 3, 4, 5, 6, 7). We examine how the environment, including diet, impacts metabolite availability and consequently epigenetic enzyme activity, but we refer the reader to more focussed reviews for detailed information of these metabolic pathways [8,9]. We particularly focus on the subcellular localisation of metabolite pools and the crosstalk between mitochondrial and nuclear proteins and RNAs. We consider some aspects of mito-nuclear communication involving histone proteins and their potential epigenetic marks (Section 8) and discuss how nuclear-encoded enzymes regulate mitochondrial function through epitranscriptomic pathways involving various classes of RNA molecules in the mitochondria (Section 9).

## 2 Histone acetylation

Histone acetylation is typically linked to chromatin decompaction and is therefore an epigenetic mark mostly associated with active transcription. Histone lysine acetyltransferases (HATs) and lysine deacetylases (HDACs) are the nuclear-encoded enzymes responsible for adding and removing histone acetyl groups, respectively. There are three main families of HATs whose enzymatic activities have been well-characterised and their biological functions determined: the MYST family, the p300 family, and the GCN5/PCAF family [3,10]. All HATs utilise acetyl-CoA as a cofactor and catalyse the transfer of an acetyl group to the ε-amino group of lysine side chains that neutralise the positive charge of the amino acid.

Numerous genome-wide studies have demonstrated that histone acetylation is sensitive to the availability of acetyl-CoA, which responds to nutrient availability or metabolic reprogramming [[11], [12], [13], [14]]. Most HATs display relatively high affinity for acetyl-CoA with K_m_ or K_d_ in the lower micromolar range (∼1 μM), indicating that most enzymes respond similarly to fluctuations in acetyl-CoA levels [15]. However, fluctuating acetyl-CoA levels significantly affect the substrate specificity of certain HATs. For example, with a limiting concentration of acetyl-CoA, p300 shows high specificity toward H4K16, while its specificity toward H4K12 is strongly reduced, at least *in vitro* [16]. In addition, a subset of HATs have the same affinity for acetyl-CoA as for coenzyme A (CoA), the by-product of the acetylation reaction. This means that CoA will compete with acetyl-CoA for enzyme binding and therefore can inhibit enzymatic activity [11]. Thus, acetyl-CoA availability is an important determinant of HAT activity. Consequently, we need to identify the various cellular pools and sites of acetyl-CoA synthesis to understand how varying levels of acetyl-CoA affect epigenetic processes. Although this review focuses on histone acetylation, RNA and many non-histone proteins are also acetylated using the same or related enzymes. As with the histones, the acetylation of these substrates is also affected by changes in the availability of acetyl-CoA.

### 2.1 Compartmentalisation of acetyl-CoA

**Figure 3:**
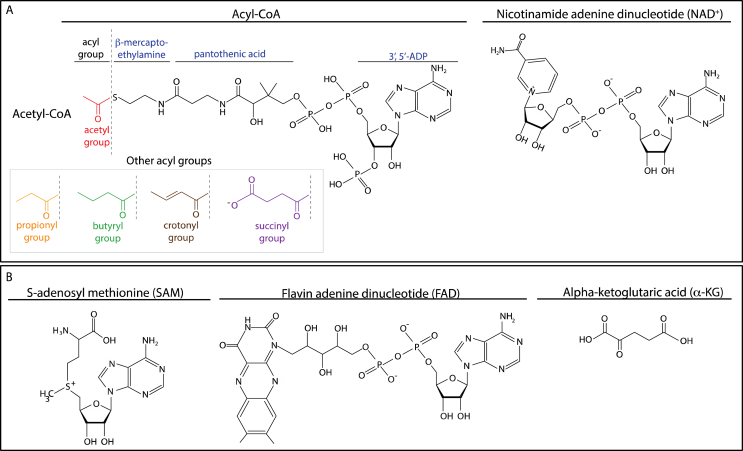
*Chemical structures of key metabolites regulating epigenetic enzymes*. **(A)** Structures of metabolites affecting histone acylation. The acetyl group donor acetyl-CoA together with other acyl moieties is depicted on the left side and the deacetylase cofactor NAD^+^ on the right side. **(B)** Structures of metabolites affecting histone and DNA methylation. The methyl group donor SAM is shown (on the left side) together with the demethylase cofactors FAD (in the centre) and α-KG (on the right side).

Acetyl-CoA is composed of an acetyl moiety linked to CoA through thioester bonds (Figure 3A). Thioester bonds are intrinsically energy rich and therefore facilitate the transfer of acetyl groups to a variety of acceptor molecules [17]. In eukaryotic cells, acetyl-CoA exists in distinct mitochondrial and extra-mitochondrial pools.

Most acetyl-CoA is generated in the mitochondrial matrix as a key metabolic intermediate of various metabolic pathways including (i) pyruvate import into mitochondria and its decarboxylation by pyruvate dehydrogenase complex (PDC), (ii) fatty acid (FA) β-oxidation, (iii) catalysis of branched chain amino acids (BCAAs, that is, leucine, isoleucine, or valine), (iv) ketone body metabolism, or (v) ligation of acetate to CoA by mitochondrial acyl-CoA synthetase short-chain family member 1 (ACSS1) [11]. Mitochondrial acetyl-CoA is fused to oxaloacetate to produce citrate that is oxidised in the tricarboxylic acid (TCA) cycle, enabling ATP production through OXPHOS.

Acetyl-CoA can be transported across mitochondrial membranes into the cytoplasm in the form of citrate. Citrate is then converted back to acetyl-CoA by ATP-citrate lyase (ACLY), which is a major source of extra-mitochondrial acetyl-CoA (Figure 2). However, its production depends on mitochondrial activity that synthesises the citrate. Cytoplasmic acetyl-CoA is an important building block for lipids, steroids, the synthesis of specific amino acids including glutamate, proline, and arginine, as well as a cofactor for HATs [11].

Citrate is a small molecule that diffuses freely through nuclear pore complexes [18]. Notably, ACLY can also translocate from the cytoplasm to the nucleus, where it can regulate global histone acetylation levels by generating a nuclear pool of acetyl-CoA as demonstrated in yeast [19], flies [20], and mammalian cells [[21], [22], [23]] (Figure 2). Upon addition of glucose to serum starved cells, nuclear ACLY facilitates histone acetylation and consequent induction of a specific set of genes involved in glucose metabolism [21]. Under conditions of glucose restriction or inhibition of glycolysis, acetyl-CoA and histone acetylation levels are suppressed [23,24]. While the growth of solid tumours heavily depends on aerobic glycolysis (known as the Warburg effect), certain cancers have developed evasion mechanisms to sustain glucose-independent acetyl-CoA production even under nutrient deprivation. For instance, phosphorylation of ACLY by AKT kinase (a signalling molecule in the insulin signalling pathway) results in enhanced enzymatic activity and increased histone acetylation followed by the expression of genes promoting cell cycle progression and proliferation [23].

Another important source of cytoplasmic acetyl-CoA is generated via the conversion of acetate to acetyl-CoA by acyl-CoA synthetase short-chain family member 2 (ACSS2) (Figure 2). It is the cytoplasmic counterpart of mitochondrial ACSS1 and uses exogenous acetate, *de novo* generated acetate from pyruvate [25], and recycled acetate produced intracellularly as a by-product of HDACs [26].

Similar to citrate, acetate diffuses freely through nuclear pore complexes [18]. ACSS2 is also found in the nucleus, where it plays a key role in certain developmental programmes. For instance, during neuronal differentiation, ACSS2 becomes nuclear and its activity is required for histone acetylation in genes involved in neuronal differentiation and consequently their expression [27]. ACSS2 interacts with the histone acetyltransferase CREB-binding protein (CBP), functioning as a chromatin-bound coactivator by locally providing acetyl-CoA, and its loss results in global reduction in histone 3 lysine 27 acetylation (H3K27ac) in neuronal stem cells [27]. Consequently, deletion of the enzyme in the mouse hippocampus, an area associated with memory formation and learning, results in defects in spatial memory [27]. ACSS2-mediated histone acetylation in the brain and the subsequent regulation of neural functions is affected by alcohol metabolism. In mice, alcohol consumption modulates gene expression in the brain and affects behaviour in an ACSS2-dependent manner, directly linking dietary habits to gene regulation [28].

PDC, which converts glycolysis-derived pyruvate into acetyl-CoA, is another well-characterised example of a mitochondrial enzyme localising to the nucleus (Figure 2). During growth factor stimulation or mitochondrial stress, PDC accumulates in the nucleus and epigenetically regulates gene expression via histone acetylation [29]. Studies of early mouse and human embryos further demonstrate that PDC components, as well as some TCA cycle enzymes, localise to the nucleus exclusively at the time of zygotic genome activation (ZGA), indicating that nuclear localisation can be tightly temporally restricted [30]. At ZGA, these mitochondrial enzymes produce key metabolites that are essential for epigenetic remodelling to enable further developmental progression [30].

Taken together, these selective studies demonstrate that cells have evolved sophisticated mechanisms to sense and integrate nutrient signals to regulate the appropriate epigenetic gene expression programmes via their control of acetyl-CoA availability (Figure 2).

### 2.2 Beyond histone acetylation: acyl-CoAs

HATs can accommodate not only acetyl-CoA, but also other acyl-CoAs such as propionyl-CoA, butyryl-CoA, crotonyl-CoA, and succinyl-CoA (Figure 3A). These acyl-CoAs serve as additional metabolic intermediates that lead to the modification of ε-lysine residues of histones. Most histone acylation sites overlap with known histone acetylation sites and are also linked to active gene expression [31]. Similar to histone acetylation, the level of acyl modification depends on the levels of the respective acyl-CoA present in cells. For instance, in mice, knockout of propionyl-CoA carboxylase alpha subunit (PCCA), an enzyme responsible for propionyl-CoA degradation, results in a global increase in propionylation of H3K14 in liver tissue [32].

Enzymes generating acyl-CoAs are mainly involved in FA β-oxidation and BCAA catabolism in mitochondria. In addition, acetyl-CoA synthetase ACSS2 can catalyse crotonyl-CoA from crotonate and its deletion results in a decrease in p300-mediated H3K18 crotonylation accompanied by reduced gene expression [33]. Thus, not only are acyl-CoAs competing for the same HATs, but enzymes involved in their biosynthesis are also shared, suggesting that regulation of histone acetylation and acylation are closely linked and respond to the same metabolic changes to initiate similar, although not identical, changes in gene expression.

Alpha-ketoglutarate dehydrogenase (α-KGDH) is another example of a mitochondrial enzyme acquiring nuclear functions. In the mitochondria, it catalyses the conversion of alpha-ketoglutarate (α-KG) into succinyl-CoA. However, in glioblastoma cells, α-KGDH partially locates to the nucleus where it interacts with histone lysine acetyltransferase GCN5 at gene promoters [34]. In this way, α-KGDH locally supplies succinyl-CoA, which boosts GCN5-mediated succinylation of H3K79, resulting in activation of genes promoting tumour cell proliferation (Figure 2) [34].

In summary, acyl-CoA levels, including acetyl-CoA, are tightly regulated by metabolic processes. This further contributes to the complex network of crosstalk between mitochondrial metabolism and epigenetic regulation in the nucleus. However, the biological functions of many histone acylation sites remain unknown and their investigation and potential metabolic regulation remains a topic of future research.

### 2.3 Non-histone acetylation

Approximately 1700 non-histone mammalian proteins have been shown to harbour at least one acetylated ε-lysine, with more than 300 of these localised in the mitochondria [35]. As many of these mitochondrial proteins are metabolic enzymes, considerable research has been devoted to determining whether HATs and HDACs, usually located in the nucleus, also function in the mitochondria. While several HDACs are known to reside in the mitochondria (discussed later in more detail; Figure 2), male on the first (MOF) was recently discovered as the first nuclear HAT to localise to the organelle in human HeLa cells [36]. Nuclear MOF is found in two distinct protein complexes, the male-specific lethal (MSL) and non-specific lethal (NSL) complexes, which are strong transcriptional activators responsible for most H4K16 acetylation [37,38]. In HeLa cells grown in galactose (not glucose) media, conditions in which cells switch to mitochondrial-based OXPHOS for ATP synthesis, MOF and a subset of its NSL complex partners are necessary for the proper expression of respiratory genes encoded in the mitochondrial genome [36]. While the exact mechanism through which MOF regulates mitochondrial transcription remains to be elucidated, it depends on the enzyme's catalytic activity [36]. A recent study that assessed global changes in the acetylome upon deletion of MOF in mammalian cells detected a range of potential mitochondrial target proteins [39].

A recent report identified mitochondrial localisation of GCN5 in yeast (Figure 2), where loss of the enzyme results in decreased mtDNA copy numbers [40]. However, whether the acetyltransferase activity of GCN5 is involved in maintaining mtDNA integrity remains an open question. However, neither MOF nor GCN5 contain classical mitochondrial targeting signals, indicating that other, yet unknown factors are involved in regulating their import into the mitochondria.

These studies raise the prospect that cells not only shuttle metabolic enzymes to meet the metabolic demand of the nucleus, but additionally utilise nuclear epigenetic enzymes as elements of communication between the mitochondria and nucleus.

## 3 Histone deacetylation

There are two main families of HDACs with distinct catalytic mechanisms, Zn^2+^-dependent histone deacetylases (HDAC1-11) and nicotinamide adenine dinucleotide (NAD^+^)-dependent sirtuin deacetylases (SIRT1-7), both of which catalyse the removal of acetyl groups from lysine side chains thereby releasing acetate. Zn^2+^-dependent deacetylases are predominantly localised in the nucleus and cytoplasm. However, HDAC1 [41] and HDAC7 [42] have been reported to reside in the mitochondria of mammalian cells (Figure 2), although their functions in the organelles are poorly understood. Sirtuins are found in the nucleus (SIRT1, 2, 3, and 7), cytoplasm (SIRT1, 2), and mitochondria (SIRT3, 4, and 5) (Figure 2). They depend on NAD^+^ as a cofactor and their activity is primarily influenced by fluctuations in NAD^+^ levels, and therefore they are sensitive to nutrient availability of the cell. Regulation of selected sirtuins by cellular metabolism are discussed in more detail to follow.

### 3.1 Compartmentalisation of NAD^+^

NAD^+^ functions as a ubiquitous electron transfer molecule in redox reactions and is found in an oxidised (NAD^+^) or a reduced form (NADH) (Figure 3A). NAD^+^ can be synthesised in two ways: (i) *de novo* from the amino acid tryptophan or via the NAD salvage pathway, which uses the NAD precursors nicotinic acid (NA), nicotinamide (NAM), or nicotinamide ribose (NR) directly from the diet, or (ii) it can be recycled from intracellular reactions (Figure 2). In the first rate-limiting step, nicotinamide phosphoribosyl transferase (NAMPT) converts NAD precursors into nicotinamide mononucleotide (NMN), which is then condensed with ATP by nicotinamide mononucleotide adenylyltransferase (NMNAT). Deletion of either of these enzymes causes lethality [43]. While NAMPT localises to both the mitochondria and nuclei [44], there are three isoforms of NMNATs, each present in a different cellular compartment: NMNAT1 (nucleus), NMNAT2 (cytosol), and NMNAT3 (mitochondria). This suggests that NAD^+^ salvage is tailored according to the metabolic needs of cellular compartments [45]. Nuclear NMNAT1 interacts with SIRT1 at gene promoters, locally providing its cofactor to promote histone deacetylation [46].

As NAD^+^ passively diffuses through nuclear pores, the metabolite concentration is equal in the nuclear and cytoplasmic compartments (∼100 μM) [47]. In contrast, when NAD^+^ diffuses freely through the outer mitochondrial membrane, the inner membrane permeability is restricted by membrane transporters. As a consequence, the mitochondrial concentration of free NAD^+^ (∼250 μM) is strictly maintained, even when NAD^+^ in the nucleus and cytosol has fallen below normal physiological levels [47]. This partitioning allows cells to maintain NAD^+^ levels under cellular stress conditions, as NAD^+^ is a central cofactor for enzymes involved in several major metabolic pathways, such as the TCA cycle, FA β-oxidation, the electron transport chain in mitochondria, and glycolysis in the cytoplasm. The central role of NAD^+^ as a metabolic intermediate directly links sirtuin activity to cellular metabolism. In turn, sirtuins directly affect the metabolic status of the cell through epigenetic mechanisms and by affecting the functions of key transcription factors regulating metabolic genes. Some examples of this interplay are presented in the following sections. For a more comprehensive discussion of sirtuin-mediated regulation of metabolism, we refer the reader to reference [48].

### 3.2 NAD^+^ and sirtuins

SIRT1 is a predominantly nuclear sirtuin regulating chromatin structure, genomic stability, and cellular metabolism (reviewed in [47]). SIRT1's dissociation constant for NAD^+^ (K_m_ = 94 μM) is very similar to the local concentration of NAD^+^ measured in the nucleus [49], and its enzymatic activity is strictly governed by fluctuations in nuclear NAD^+^ levels. For example, certain dietary conditions such as caloric restriction (CR) can trigger upregulation of NAMPT, the rate-limiting enzyme in NAD^+^ biosynthesis (as previously discussed). Increased NAD^+^ levels activate SIRT1, which improves metabolic homeostasis through different mechanisms depending on the metabolic demand of the tissue [8]. A main target of SIRT1 is peroxisome proliferator-activated receptor gamma coactivator 1 alpha (PGC-1α), a key metabolic coactivator of transcription factors regulating mitochondrial homeostasis and metabolic gene expression. To adapt to CR, SIRT1-mediated deacetylation of PGC-1α triggers the activation of genes enabling gluconeogenesis and hepatic glucose output in the liver [50], as well as activation of mitochondrial FA β-oxidation genes in the skeletal muscle [51]. In a similar fashion, SIRT7-mediated deacetylation of GA-binding protein transcription factor subunit beta (GABPβ) facilitates transcriptional activation of the GABPβ/GABPα heterodimer, a master regulator of nuclear-encoded mitochondrial genes [52]. Consequently, *Sirt7* knockout mice develop multisystemic mitochondrial dysfunction, particularly in mitochondria-heavy tissues such as the liver [52].

SIRT6 is a major epigenetic regulator of glucose metabolism as it catalyses deacetylation of H3K9 and H3K56 to repress expression of hypoxia inducible factor 1 subunit alpha (HIF1α)-driven glycolytic genes [53]. *Sirt6* gene knockout is lethal in mice due to fatal hypoglycaemia caused by a constant upregulation of glycolysis and an uncontrolled uptake of glucose by specific tissues such as brown fat [53]. In comparison to other sirtuins, SIRT6 binds NAD^+^ with high affinity, suggesting its activity is less affected by fluctuations in NAD^+^ levels.

Although most SIRT3 is found in the mitochondria, it is also present in the nucleus. The full-length form of human SIRT3 (38 kDa) exists in the nucleus. It is processed to generate a distinctly shorter form (28 kDa) that then localises to the mitochondria [54]. Both forms harbour deacetylase activity [55]. ChIP-sequencing experiments analysing full-length human SIRT3 revealed its association with genes involved in stress response, lipid metabolism, and mitochondrial function [55]. Although SIRT3's histone substrates are not well characterised, it has been reported to deacetylate H4K16ac *in vitro* and *in vivo* using reporter assays [54]. Full-length SIRT3 has been shown to relocate from the nucleus to mitochondria under stress conditions [54], where it plays a critical role in responding to oxidative and metabolic stress [56,57].

While its function in the nucleus is only partially understood, SIRT3's mitochondrial targets are much better characterised. In contrast to knockout of the other mitochondrial sirtuins (SIRT4 or 5), loss of SIRT3 in mice results in global hyperacetylation of numerous mitochondrial proteins [58]. While under basal conditions, *Sirt3* knockout mice are metabolically normal [58], mitochondrial hyperacetylation significantly increases susceptibility to metabolic syndromes such as obesity or diabetes in response to high-fat treatment [59]. SIRT3-mediated regulation of mitochondrial function in mice has been best-characterised in mitochondria-rich tissues such as the liver, where it modulates the enzymatic activities of proteins involved in FA β-oxidation, OXPHOS, ketone body synthesis, and the urea cycle [60,61]. SIRT3 targets include key metabolic enzymes such as ACSS1 [62,63], PDH [64], isocitrate dehydrogenase 2 (IDH2) [65], glutamate dehydrogenase 1 (GDH1) [58], succinate dehydrogenase flavoprotein (SDHA) [56], and NADH ubiquinone oxidoreductase subunit A9 (NDUFA9) [66]. Notably, some of these mitochondrial targets are known to localise to both the mitochondria and nucleus such as the previously discussed PDH [29,30] or GDH1 [67].

In the mitochondria, GDH1 is a key enzyme in glutaminolysis converting glutamate and NAD^+^ into α-KG, thereby providing a TCA cycle intermediate metabolite as well as a cofactor for histone demethylases and DNA hydroxylating enzymes. Nuclear GDH1 was reported in chicken liver tissue to bind chromatin and has H3 N-terminal tail-clipping activity [67,68], a mechanism known to regulate gene expression [69,70]. Thus, GDH1 is another candidate linking mitochondrial metabolism and nuclear gene regulation. Whether it is the same pool of GDH1 that shuttles between the two organelles and what exactly governs the switch between its dehydrogenase or serine–protease activity remain open questions.

In summary, to date SIRT3 is the only sirtuin that localises to both the mitochondria and nuclei. Because full-length SIRT3 is cleaved in the nucleus before entering the mitochondria, SIRT3 communication between the nucleus and mitochondria is most likely unidirectional and has to date been reported only under cellular stress conditions. In the mitochondrial matrix, it is regarded as a key metabolic sensor that modulates the activity of numerous metabolic enzymes, while in the nucleus, it regulates genes involved in stress response and mitochondrial lipid metabolism. Therefore, SIRT3 strongly links mitochondrial metabolism and epigenetic gene regulation.

## 4 Histone methylation

Histones may be methylated to different degrees: lysines (Ks) may be mono-, di-, or trimethylated, while arginines (Rs) may be mono-, symmetrically, or asymmetrically dimethylated [2]. The best-characterised sites of histone methylation are lysines, and therefore this section focuses on methylation pathways targeting histone lysines.

Methylation does not affect the overall ionic charge of chromatin but rather serves as a docking site for chromodomain-containing epigenetic readers. Unlike acetylation, histone methylation is associated with both repression and activation of transcription, depending on the residue, the number of methyl groups, and the actual histone modified. For example, methylation of H3K4 and H3K36 is associated with active genes, whereas methylation of other residues such as H3K9, H3K27, and H4K20 is associated with transcriptional repression.

Histone lysine methyltransferases (HKMTs) and histone lysine demethylases (HKDM) are the enzymes responsible for transferring or removing a methyl group from lysine ε-amino side chains, respectively. Most HKMTs contain a su(var)3–9, enhancer-of-zeste, and trithorax (SET) catalytic domain and all utilise S-adenosylmethionine (SAM) as a cofactor. SAM is a metabolic intermediate serving as a universal high-energy donor for methyl groups to all methylating enzymes, including those that target DNA, histones, and RNA (Figure 3B). The cofactor is produced in the cytoplasm from methionine and ATP by the enzyme S-adenosyl methionine transferase (MAT), a reaction that is part of the one-carbon cycle describing the conversion of homocysteine into methionine (Figure 2) [71]. The one-carbon cycle is tightly linked to the diet as it relies on the action of numerous enzymes that require dietary micronutrients such as folate and betaine for their catalysis.

Under basal conditions, cellular concentrations of SAM are similar to HKMT K_m_ values (K_m_ ∼1–10 μM), suggesting that HKMTs are sensitive to fluctuating concentrations of SAM [72]. Knockdown of SAM synthetase isoform MAT2A results in global reduction in H3K9me3 and H3K4me3 levels in mammalian cells [73]. MAT2A localises to the nucleus, where it interacts with HKMT SET domain bifurcated histone lysine methyltransferase 1 (SETDB1) at chromatin and inhibits *Cox2* gene expression by providing a local pool of SAM [73]. The expression of MAT2A is sensitive to the intracellular concentration of SAM via mechanisms affecting its mRNA [74,75], generating a feedback loop controlling the abundance of the enzyme in relation to SAM availability.

In addition to changes in SAM levels (either locally or globally), HKMTs can be regulated by S-adenosyl-homocysteine (SAH), a by-product of methyltransferase reactions known to inhibit HKMT activity (Figure 1) [76]. SAH is further hydrolysed to homocysteine, which is recycled back to methionine using 5-methyltetrahydrofolate (5-MTHF) as a methyl donor that can be derived from dietary folic acid. Therefore, although generated in the cytoplasm, SAM synthesis and recycling through the one-carbon cycle directly depends on the folate cycle as well as ATP, both of which rely on mitochondrial activity. Also, significant changes in methionine uptake through the diet lead to fluctuations in one-carbon metabolism and SAM levels, which ultimately results in changes in H3K4me3 and gene expression as shown in mice [77]. Overall, these results indicate a direct communication between sensing methionine availability and chromatin regulation.

## 5 DNA methylation

DNA methylation modifies chromatin structure and silences gene expression by changing the structure of a single nucleotide. In eukaryotes, 5-methylcytosine (m5C) is the most abundant and most studied DNA modification. However, other modifications such as oxidation of m5C to 5-hydroxymethylcytosine (hm5C), 5-formylcytosine (f5C) and 5-carboxylcytosine (ca5C), and methylation of adenine (A) to N^6^-methyladenine (m6A) have been identified and their significance as epigenetic regulators is becoming increasingly apparent [78]. In mammals, m5C is typically found on CpG sites that are often clustered in regions known as CpG islands. m5C-mediated gene silencing is involved in numerous biological processes such as genomic imprinting, epigenetic memory, and X chromosome inactivation. DNA and histone methylation are tightly linked and have synergistic effects on regulating gene expression.

Three DNA methyltransferases (DNMT1, DNMT3A, and DNMT3B) have been identified in humans. DNMT1 is a maintenance methyltransferase that mainly functions after DNA replication to copy the methylation pattern of the parental strand. DNMT3A and DNMT3B function predominantly as *de novo* methyltransferases to establish DNA methylation during embryogenesis [4]. DNMTs also use SAM as a methyl donor, so the enzymes directly compete with protein methyltransferases for their cofactor and are equally affected by fluctuations in the one-carbon and folate cycles, which intrinsically link DNA methylation to cellular metabolism and dietary factors.

In a cell culture system of induced mtDNA depletion, early transcriptional changes in genes responding to mitochondrial dysfunction are detected, accompanied by increased DNA methylation at their promoters [79]. Increased DNA methylation is linked to acute enhancement of methionine metabolism, largely via the methionine salvage pathways, and increased production of SAM in these cells [79]. In the same system, at the same timepoint after induction of mtDNA depletion, histone methylation remains unaffected [80], indicating that during mitochondrial dysfunction, DNMTs respond more quickly to fluctuations in SAM levels than histone methyltransferases.

Many nuclear-encoded mitochondrial protein genes are regulated by DNA methylation in response to metabolic changes. For example, in the skeletal muscle of type 2 diabetes patients, expression of the *PGC-1α* gene, which encodes a key modulator of transcription factors regulating mitochondrial biogenesis, is repressed by DNMT3B-mediated hypermethylation in response to free fatty acids and inflammatory signals, resulting in reduced mitochondrial density [81].

Although DNMT1 has also been found to reside in the mitochondria [82], mtDNA methylation has been debated over many years with numerous datasets either supporting or rejecting the presence of methylated sites in the mitochondrial genome [83,84]. If at all present, the function of mtDNA modifications and whether they employ similar epigenetic mechanisms to regulate mitochondrial gene expression remain elusive.

While a direct DNA demethylase has yet to be discovered, DNA methylation can be reversed through stepwise oxidation reactions. Ten-eleven translocation (TET) enzymes are responsible for oxidising m5C to 5-hydroxy-methylcytosine (hm5C) and other oxidised methylcytosines, which in turn triggers a demethylation process. TET enzymes are discussed further in Section 7.

## 6 Histone demethylation

Similar to HATs and HDACs, histone demethylases (HDMs) also require metabolic intermediates as cofactors, whose synthesis depends on mitochondrial activity. There are two classes of conserved families of HDMs, the lysine-specific histone demethylase (LSD) and Jumonji C domain-containing protein (JMJDs) families, both intrinsically linked to cellular metabolism.

### 6.1 FAD and LSDs

LSDs demethylate lysines via an amine oxidation reaction with flavin adenine dinucleotide (FAD) as a cofactor (Figure 3B). FAD is generated *de novo* in the mitochondria and cytoplasm from the vitamin riboflavin (vitamin B2) by the ATP-dependent enzymes riboflavin kinase and FAD-synthase (FADS). Riboflavin is an essential metabolite that has to be supplied by the diet. FADS and FAD hydrolysing enzyme FAD pyrophosphatase are also present in the nucleus, thereby providing a nuclear pool of FAD by creating an equilibrium between FAD/FADH_2_, as occurs in the mitochondria [85]. In the mitochondria, FAD is used as a cofactor for enzymes catalysing a variety of redox reactions including oxidation, reduction, and dehydrogenase reactions. For example, acyl-CoA dehydrogenases (ACADs) catalyses the first step in FA β-oxidation or succinate dehydrogenase (SDH) complex catalysing oxidation of succinate to fumarate in the TCA cycle, where it is reduced to FADH_2_. The two high-energy electrons from FADH_2_ are used within the electron transport chain to produce ATP by OXPHOS, reverting FADH_2_ back to FAD.

In addition to the three aforementioned metabolic intermediates acetyl-CoA, NAD^+^, and SAM, FAD is a metabolic biosensor that directly influences epigenetic regulation by modulating LSD activity. Loss of riboflavin kinase or FADS in adipocytes inhibits LSD1 activity, resulting in increased H3K4 methylation, allowing master regulator PGC-1α to activate the expression of genes related to energy expenditure and ultimately mitochondrial respiration and induction of lipolysis [86]. However, white adipose tissue (WAT), which is known to enhance mitochondrial biogenesis in response to cold stimuli, upregulates LSD1 protein levels [87]. LSD1 cooperates with nuclear respiratory factor 1 (NRF1) to activate genes involved in OXPHOS to induce mitochondrial activity [87]. Taken together, LSD1 activity is regulated by metabolic and environmental cues, largely dietary in nature, and functions as an important repressor and activator of nuclear-encoded mitochondrial genes.

### 6.2 α-KG and JMJDs

The JMJD family of demethylases belongs to the α-KG-dependent dioxygenase enzymes. Their function depends on iron (Fe^2+^) and α-KG as cofactors (Figure 3B). α-KG is a key intermediate of the TCA cycle that is generated from isocitrate by isocitrate dehydrogenases 2 and 3 (IDH2 and IDH3) in the mitochondria and by IDH1 in the cytoplasm. Hence, it is apparent that deregulation of the TCA cycle or dysfunction of the mitochondria directly affects α-KG levels and consequently JMJD activity [88]. IDH3 is one of the TCA cycle enzymes that was shown to relocate to the nucleus at ZGA in early mouse and human embryos to locally provide metabolites allowing epigenetic remodelling [30] (Table 1).

The TCA cycle intermediates fumarate and succinate are potent JMJD inhibitors (Figure 1) and many cancers harbour mutations in genes encoding fumarate hydratase (FH) and/or the SDH complex, resulting in increased levels of both metabolites, leading to hypermethylation of histones [89]. Another potent inhibitor of α-KG-dependent dioxygenases is the onco-metabolite 2-hydroxyglutarate (2-HG). Cancer-associated mutations in *IDH1* and *IDH2* detected in a wide range of human cancers [90] can result in neomorphic enzymes producing 2-HG, instead of α-KG, resulting in global epigenetic changes due to inhibition of JMJDs as well as TET enzymes [90]. Furthermore, in cells lacking *IDH* mutations, cytotoxic stress conditions can promote many metabolic enzymes to overproduce 2-HG, causing pathologically significant concentrations. For example, under hypoxic conditions, both malate and lactate dehydrogenases produce 2-HG [90]. Moreover, JMJD histone demethylase activity can directly depend on oxygen concentrations in the cell. KDM5A and KDM6A were recently identified to be oxygen sensors, and changes in oxygen concentration directly modulate H3K4me3 and H3K27me3 levels [91,92].

While JMJDs are nuclear proteins and not yet known to localise to the mitochondria, their activity and genomic targets are directly regulated by factors that shuttle between the organelles. During mitochondrial depolarisation, when the membrane potential changes from negative to positive in the depolarising direction from the resting potential, the mitochondrial G-protein pathway suppressor 2 (GPS2) translocates rapidly to the nucleus, where it, together with other transcription factors and JMJD2/KDM4A, activates transcription of genes involved in the mitochondrial stress response [93]. Another important pathway to ensure mitochondrial homeostasis under stress conditions is the mitochondrial unfolded protein response (UPR^mt^), which, in worms, is a central mechanism to extend lifespan [94]. *C. elegans* jmjd-1.2 and jmjd-3.1 and their mammalian homologues PHF8 and JMJD3 have been identified as indispensable regulators of this pathway by removing repressive H3K27me2/3 marks at genes required for the UPR^mt^ response [94]. However, how nuclear PHF8 and JMJD3 sense mitochondrial dysfunction in the first place and whether this is achieved through mito-nuclear shuttling of an unknown factor or through fluctuations in α-KG levels remains to be determined.

## 7 DNA demethylation

The TET family of enzymes (TET1, 2, and 3) are methylcytosine dioxygenases that catalyse the oxidation of m5C to hm5C and promote locus-specific reversal of DNA methylation. hm5C can either trigger base excision repair (BER) to exchange hm5C to an unmethylated cytosine or it can be the substrate of further TET-mediated oxidation reactions that form f5C and ca5C, all of which can function as epigenetic determinants regulating gene expression (reviewed in [95]). In addition to DNMT1, TET1 and TET2 have also been found in the mitochondria (Figure 2) [96]; however, the existence, let alone the function, of hm5C on mtDNA remain a subject of much debate [83].

Analogous to JMJDs, TET activity depends on α-KG and Fe^2+^ (Figure 1). Moreover, both enzyme families are inhibited by fumarate, succinate, and 2-HG. Therefore, TCA cycle deregulation affects their activity and consequently DNA methylation. Both DNMT and TET activity is strongly influenced by environmental exposure to organic pollutants, tobacco smoke, and nutrition resulting in altered global and gene-specific DNA methylation. For a complete discussion of how environmental cues affect the epigenome, we refer the reader to a recent review [97].

## 8 Communication involving histones and their marks

Histones are the canvas upon which most epigenetic information is recorded and interpreted. Thus, they are the central components of all epigenetic pathways. While it is clear that mitochondrial genomes are not compacted by histones, it is evident that histones, and therefore potentially their epigenetic marks, play a direct role in mitochondrial processes. At the most basic level, epigenetic modifications control the transcription of nuclear genes whose products, as RNA or protein, can translocate to the mitochondria to exert their influence. In this section, we present a brief overview of how histones function and how their modifications have been directly linked to mitochondrial function.

The levels of histone proteins are important regulators of mitochondrial function. In yeast, reduced histone production stimulates mitochondrial activity leading to increased production of ATP [98]. *In addition, H2AX* mRNA is targeted for degradation by a miRNA, *miR-24*, and the consequent depletion of the histone negatively affects mitochondrial function and has been implicated in the pathogenesis of insulin resistance [99]. H2AX is actually a histone variant protein that is phosphorylated by the ATM kinase to initiate double-strand DNA break repair. Interstingly, mitochondria in the brain are uniquely susceptible to damage in H2AX mutant mice and H2AX maintains neuronal health via the regulation of mitochondrial homeostasis [100]. This control is mediated, at least in part, by H2AX control of an NRF2-regulated antioxidant response pathway and regulating the expression of the gene encoding PGC-1α [100,101]. Considered together, these findings demonstrate that compromised redox homeostasis due to mitochondrial defects is often linked to neurological pathologies [[101], [102], [103]]. They also reinforce how changes in the nuclear genome can trigger changes in mitochondrial activity and cell metabolism.

H2AX might also play a more direct role in regulating mitochondrial activity because it has been reported to be present in the mitochondrial outer membrane. The role of H2AX in this location appears to be independent of DNA damage repair as ionising radiation (that induces DNA double-strand breaks) did not lead to detectable phosphorylation of H2AX within mitochondria [104]. It may function in the process of transporting proteins into the mitochondria in a transcription-independent manner [104].

Other histones have also been associated with mitochondria. For example, released H3 hypo-phosphorylated at serine 10 is translocated to the mitochondria in certain human cancer cells during apoptosis (Figure 2) [105]. Another example involving histone translocation is observed in T-effector cells following the induction of apoptosis by cytokine withdrawal. This promotes leptomycin B (LMB)-sensitive nuclear export of linker H1.2 from the nucleus to mitochondria (Figure 2) [106]. There, it associates with the mitochondrial apoptotic protein BAK to trigger T-effector cell apoptosis [106]. The translocation of H1.2 is affected by changes in the cellular acetylome (all acetylated proteins), raising the possibility that epigenetic modifications, perhaps of H1.2 itself, may play a role in the process.

Neutrophils are an important part of the innate immune response and represent 60–70% of all white blood cells. They are among the first responders to sites of injury and use multiple strategies to fight pathogens. One strategy is neutrophil extracellular trap (NET) formation (the process is called NETosis) where, in response to specific inflammatory stimuli, neutrophils undergo programmed cell death involving de-condensation of their chromatin (coated with cytotoxic peptides) and its extrusion from the cell. This is an epigenetically controlled pathway requiring histone citrullination, preferentially in gene promoters by the peptidyl arginine deiminase 4 (PADI4) enzyme [107]. The mitochondria play a role in NETosis because one of the major pathways through which NETs are formed requires mitochondrial ROS production [108]. Furthermore, ATP derived from the mitochondria is required to produce the microtubule network that supports NET formation [109]. Histone acetylation also promotes NET formation by facilitating chromatin decompaction. Thus, NETosis represents a component of the immune system that requires the coordinated action of multiple histone epigenetic modification enzymes and mitochondrial activity.

## 9 RNA modification pathways in mitochondria

In a similar fashion to histones, RNA molecules are also subject to covalent modification during or following their synthesis. To date, well over 150 modifications have been detected on RNAs, and the number continues to increase. They are present on every class of RNA and are evolutionarily conserved throughout all kingdoms of life [110,111]. Together, they are called the “epitranscriptome” and have the potential to regulate all RNA-dependent functions and activities. However, all RNA-modifying enzymes are nuclear encoded and specific enzymes have to be trafficked to the mitochondria, providing a direct mechanism for nucleus/mitochondria communication (Figure 2). As with the previously discussed enzymes, their catalytic activity is linked to the metabolic status of the cell, which in turn can be affected by environmental factors such as diet.

The mitochondrial genome encodes multiples classes of RNA molecules, mitochondrial-rRNA (mt-rRNA), mt-tRNA, mt-mRNA, mt-microRNA (mt-miRNA), and mt-long non-coding RNA (mt-lncRNA). Of these, there are 22 mt-tRNAs and 2 mt-rRNAs, and 13 mt-mRNAs that encode proteins that function in the OXPHOS pathway. The tRNAs contribute to both mitochondrial and cytoplasmic translation processes, whereas the rRNAs are unique to mitochondrial translation. The communication between the nucleus and mitochondria is even more complex given that nuclear-encoded tRNA-modifying enzymes, such as pseudouridine synthase 1 (PUS1), modify both mitochondrial and cytoplasmic RNAs. Thus, crosstalk between mitochondrial protein synthesis and cytoplasmic protein synthesis machineries exists [112]. This level of communication must underlie the mechanisms through which the mitochondrial and cytoplasmic translation machineries are coordinated to ensure, for example, how cytoplasmically translated components of the OXPHOS system are synthesised equally to those translated by the mitochondrial ribosomes [113].

The next section briefly describes how RNA modifications and their associated enzymes are used to communicate information between the nuclear genome and mitochondria (and vice versa). Although many, if not most, RNA modifications occur within the cytoplasm, including the modification of RNAs exported from the mitochondria, the enzymes mediating these reactions are affected by cofactor availability as already discussed in detail. The next section therefore focuses on communication mediated by nuclear-encoded RNA-modifying enzymes within mitochondria as this facilitates additional levels of control.

### 9.1 Modification of miRNAs

In eukaryotes, miRNAs are short non-coding single-stranded RNA molecules (18–24 nucleotides) that post-transcriptionally inhibit gene expression by targeting the RNA interference silencing complex (RISC) to specific mRNAs. Recognition of specific mRNAs is achieved via partial base pairing to sequences in mRNAs, predominantly in 3′ UTRs [114]. This inhibits translation of the encoded proteins and/or leads to degradation of the mRNA targets [115,116]. Over 1,000 miRNAs have been identified in humans, and they regulate many important physiological and pathological processes.

miRNA biosynthesis occurs via a multistep pathway that can be regulated by various mechanisms [117], including post-transcriptional modification of precursor miRNAs. Following their transcription, primary miRNA transcripts (pri-mRNA) are cleaved by the DROSHA nuclease to yield pre-miRNAs, which are then cleaved by the DICER nuclease into mature double-stranded miRNAs. Modifications that regulate their biosynthesis include methylation of the 5′ monophosphate of pre-miRNAs, m6A methylation of pri-miRNAs, and m7G methylation of G-quadruplex structures in pri-miRNA [[118], [119], [120]]. These epitranscriptomic modifications, and other yet to be identified changes, also have the potential to regulate the activity of the miRNA in all downstream processes.

Numerous miRNAs have been found enriched in the mitochondria (reviewed in [121]) and they originate from genomic and also likely mitochondrial genes. Their identification raises many questions, such as is their biosynthesis regulated by modifications, and do modifications by nuclear-encoded enzymes regulate their function or target recognition? In addition, for nuclear-encoded miRNAs, there is the question of how they are trafficked to the mitochondria and whether modifications affect this (Figure 2). Although these are difficult questions to address, some information is available.

Numerous miRNAs originating in the nucleus have been reported to target mt-mRNAs, highlighting the potential for communication between the two cellular compartments (see [122]). For example, during muscle development, *miR-1* translocates into the mitochondria and targets the mitochondrial cytochrome c oxidase subunit 1 (MT–CO1) and NADH:ubiquinone oxidoreductase core subunit 1 (*Nuf4a*) mRNAs [123]. Furthermore, it has reported that a number of *let-7* family members are enriched in the mitochondria in most cell types [121], where *let-7a* and *let7b* have multiple sites of interaction in the mitochondrial genome. These include both coding and non-coding areas, the latter of which include hypervariable regions [124]. Importantly, the biosynthesis of the *let-7* family of miRNAs is facilitated by METTL1 methyltransferase deposition of m7G within a quadruplex structure, as it promotes processing of the pri-miRNA [120]. The pri-miRNAs of other mitochondria-associated miRNAs, such as *miR-*204 that targets *LC3-II* mRNA and regulates cardiomyocyte autophagy [125], are also predicted to contain a G-quadruplex structure (unpublished data), raising the possibility that their biosynthesis is also regulated by METTL1-mediated m7G methylation. It is also notable that METTL1 activity is regulated by phosphorylation via the AKT pathway linking its activity, and therefore the biosynthesis of various miRNAs, to metabolic control (that is, the insulin pathway) [126].

### 9.2 Modification of tRNAs

The 22 mt-tRNAs are essential for mitochondrial mRNAs to be translated by mitochondrial ribosomes. In turn, this translational activity is essential for mitochondrial function because these ribosomes translate mitochondrially encoded polypeptides essential for the OXPHOS process [112]. The tRNAs are generated from a precursor transcript by two mitochondrial RNases, P and Z, which generate their 5′ and 3’ ends, respectively.

Per unit length, tRNAs are undoubtedly the most heavily post-transcriptionally modified class of RNA. This is notable because the enzymes that catalyse the modifications are nuclear-encoded, cytoplasmic-translated, and imported into the mitochondria, whereupon they modify tRNAs to regulate their function, predominantly in protein translation. However, there are far too many tRNA modifications to discuss in detail here, so we will briefly consider some of the implications and refer the reader to an excellent review of this field [112].

The plethora of tRNA modifications can be broadly split into two demographics, those in and immediately surrounding the anticodon loop and those in the tRNA core. Anticodon modifications regulate decoding capacity and overall translational accuracy, whereas core modifications predominantly affect tRNA stability [112]. The importance of these modifications is underscored by the fact that mutations of many of the relevant enzymes have been tightly linked to mammalian disease. For instance, mutations of the pseudouridylase PUS1, which isomerises uridine 27 and 28 of certain tRNAs to pseudouridine, lead to mitochondrial myopathy, lactic acidosis, and sideroblastic anaemia [127,128]. Similarly, mutations of the tRNA methyltransferase 5 (TRMT5), which methylates G37 within the anticodon loop of specific tRNAs, lead to numerous pathologies including mitochondrial myopathy and lactic acidosis [129]. Considering all of these factors together, it is clear that the production and transport of nuclear-encoded key tRNA-modifying enzymes and the resulting modification of tRNAs within the mitochondria play a critical role in regulating mitochondrial activity. Moreover, the mitochondrial genome does not encode a full complement of tRNAs and consequently those missing need to be imported from the cytoplasm using a mechanism linked to the protein importation pathway. Thus, cytoplasmic availability and the ability to import nuclear-encoded tRNAs into the mitochondria represent additional regulatable steps in the coordination of cellular translation.

### 9.3 Modification of rRNAs

The mitochondrial ribosome (mito-ribosome) is composed of two subunits, a large 39S subunit and a small 28S subunit that contain mitochondrially encoded *16S* and *12S* rRNAs, respectively. Each subunit also contains a number of ribosomal proteins (52 and 30 for large and small, respectively). As with mt-tRNAs, all modifications of mt-rRNAs are catalysed by nuclear-encoded-modifying enzymes, and again their importance is highlighted by the findings that mutations of the enzymes’ genes are associated with human disease. For instance, loss of mitochondrial transcription factor B1 (TFB1M), which catalyses m^6^_2_A methylation of A936 and A937 in *12S* rRNA, has been linked to type 2 diabetes and mitochondrial-associated deafness, presumably because the modifications regulate the translational efficiency of the mito-ribosome (for a full review, see [112]).

Although far fewer modifications of the mt-rRNAs have been detected compared to their cytoplasmic counterparts, at least one of the modified nucleotides is not conserved in cytoplasmic rRNA. More specifically, A947 of human *16S* rRNA is m1A methylated by tRNA methyltransferase 61B (TRMT61B) but there is a uridine at this position in the equivalent cytoplasmic rRNA [[112], [130]]. m1A at A947 is believed to be required for optimal translation of the mito-ribosome, whereas an unmodified uridine confers the same property to cytoplasmic rRNA. Thus, the mito-ribosome has evolved to allow control of its activity by nuclear-encoded TRMT61B. However, nuclear control of mitochondrial translation is even more complex because TRMT61B also methylates mt-tRNA at position 58 to regulate its activity. Thus, m1A modification of mt-tRNA and -rRNA by a single methyltransferase may provide a level of coordinated control over mitochondrial translational activity [[112], [130]].

### 9.4 Modification of lncRNAs and mRNAs

Although the epitranscriptomes of tRNAs and rRNAs are relatively well documented, there are far fewer data concerning mRNA and lncRNA modification within mitochondria. Nevertheless, given what happens to their cytoplasmic counterparts, we believe it is highly likely that these RNAs will also be abundantly modified with mitochondria. The advent of better detection and sequencing strategies makes this an immediately testable prediction.

It was recently reported that mt-mRNAs are indeed modified (fully reviewed in [131]). For instance, pseudouridine has been identified in mt-mRNAs *MT–CO1* and *MT–CO3*, and its levels are reduced by depletion of RNA pseudouridine synthase D3 (RPUSD3) and TruB pseudouridine synthase family member 2 (TRUB2) [[132], [133], [134]]. Evidence is also emerging that mt-mRNAs are m1A-modified [135,136], although the two studies provide m1A maps with limited overlap. However, one mRNA, *MT–ND5*, was identified by both studies and it appears that the modification is catalysed by TRMT10C [136]. Mutation of *MT–ND5* is linked to mitochondrial disease and Leber's hereditary optic neuropathy but it remains to be determined whether m1A plays a role in the disease process. It is noteworthy that TRMT10C also catalyses m1A in certain mt-tRNAs to stabilise them by preventing the interaction of A9 with U64 in the t-stem [137]. Thus, once again a nuclear-encoded RNA methyltransferase modifies different RNA classes, thereby providing a mechanism to co-ordinately regulate mitochondrial activity.

## 10 Conclusions

We have discussed how the levels of metabolites are regulated between mitochondria and nuclei by transporting metabolite intermediates as well as the enzymes involved in their catalysis (Figure 2). This has a direct and profound influence on histone and RNA modifications and ultimately gene expression.

Mitochondria and nuclei share a number of common features, such as both being membrane-bound organelles containing actively transcribed DNA. However, their genomic architectures are very different, with nuclear DNA being heavily compacted into chromatin. However, not all chromatin is the same, and distinct chromatin environments exist within the nucleus, with many regions being made geographically distinct via a process called liquid–liquid phase separation (LLPS) [138]. This produces chromatin scaffold-independent liquid-like protein droplets that constrain and regulate chromatin effectors as well as the availability of their cofactors, thereby ultimately controlling gene transcription (and other DNA processes).

Many chromatin-associated molecules have been implicated in the establishment and/or maintenance of LLP-separated domains. These include (i) heterochromatin protein 1 (HP1) that specifically interacts with heterochromatic histone H3 methylated at lysine 9 (H3K9me) [139,140], (ii) lncRNA *Xist* that is essential for appropriate silencing of an X chromosome in mammalian females [141], and (iii) multiple RNA-binding proteins [142]. Post-translational and epitranscriptomic modifications are also heavily implicated in these processes: HP1 requires phosphorylation to initiate LLPS [139,140], *Xist* requires m6A to target the X chromosome [143], multiple m6A modifications within mRNAs provide binding sites for YTH domain-containing family proteins and enhances their potential to drive LLPS [144], and the activities of multiple other RNA-binding proteins are regulated by arginine methylation [142]. Thus, the availability of metabolites such as ATP, acetyl-CoA, and SAM, whose concentrations can vary depending on dietary intake, is tightly linked to the partitioning of genomic regions via LLPS and the associated effects on gene expression. However, the very presence of phase-separated domains affects the local concentrations of metabolites due to molecules in phase-separated regions with distinct diffusion coefficients. Therefore, a feedback mechanism appears to exist where the establishment, and perhaps the maintenance, of LLP-separated domains depends on the local concentration of metabolites, but once formed, the domains affect the local concentration of the same metabolites.

These examples demonstrate that RNAs play a central role in LLPS. Numerous RNA-rich “compartments” exist within cells, such as nucleoli, paraspeckles, and stress granules. However, how these regions are physically separated is not understood because they are devoid of membrane structures. It is therefore tempting to speculate that they occupy distinct and definable spaces within cells due to RNA-directed LLPS. If so, metabolite availability will likely regulate this activity, once again highlighting the complex coordination between mitochondrial and nuclear activities. Furthermore, whether such phase separations occur within mitochondria remains highly speculative but as noted, all of the necessary components for LLPS are present within these organelles. Further research is necessary to understand how they function together to drive mito-LLPS.
